# Supplementary material for: Patterns of Intron Gain and Loss in Fungi
Source: PLoS Biol. 2004 Nov 30;2(12):e422. doi: 10.1371/journal.pbio.0020422 (PMC532390; doi:10.1371/journal.pbio.0020422)
Supplement: Table S1 — Also available at http://genes.mit.edu/NielsenEtAl/. (4.3 MB ZIP). [file pbio.0020422.st001.zip › NielsenEtAl/html/1256.html]

AN0124.1.NCU07722.1.MG07119.1.FG09784.1


```
 CLUSTAL W (1.82) Multiple Sequence Alignments - Introns Inserted


Sequence 1: NCU07722.1	427 aa
Sequence 2: FG09784.1	401 aa
Sequence 3: MG07119.1	422 aa
Sequence 4: AN0124.1	405 aa
Alignment Length: 435 aa
Number Identitical Residues: 200 aa
Alignment Score (without introns) 9595


MG07119.1 	MKLDSKAMRHLTSEDWKVLTG0VEQGSKNHEIVPTPLIEKLSRLRGGASGVHRSISNLAK
NCU07722.1	MFLDTRVLRHLTAEDWRVLTA~VEMGSKNHELVPTPLIERIARLKGGASGVHKSIATLAK
FG09784.1 	MKLDTKAMRYLTDEDWRVLTA0AEMGSKNHELVPTTLIEKLAHLRGGAGSVHRSISALAK
AN0124.1  	MKLDPKAIRYLTSEDFRVLAA0VEQGSKNHEVVPTPLIAQISGLRGGS-GVNRAISNLAK
          	* **.:.:*:** **::**:. .* ******:***.** ::: *:**: .*:::*: ***

MG07119.1 	VGLIARVKEAKY~DGYRLTYGGLDYLALHTHSTKKAIYSVGTRIGVGKESDIMIVADDTG
NCU07722.1	SGLIGRMKEAKY~DGYRLTYGGLDYLALHTHSSKDVVYSVGSRVGVGKESDIMIVADKTG
FG09784.1 	VGLIARVKEAKY~DGYRLTYGGLDYLALHTHAKRNDIYSVGDRVGVGKESDIMIVADHTG
AN0124.1  	TNLIAKVKNAKY1DGYRLTYGGLDYLALNAHQKQKCIYSVGNQIGVGKESDIIVVANHEG
          	 .**.::*:*** ***************::* .:. :**** ::********::**:. *

MG07119.1 	CQRVLKIHRLGRISFRTVKANRDYLKKRGQASGSWMYLSRLAAMKEFAFMQALREEGFPV
NCU07722.1	KQKILKIHRLGRISFRTVKSNRDYLRNR--QGGSWMYLSRLAAMKEYAFMQALQEEGFPV
FG09784.1 	TQRILKIHRLGRISFRSVKSNRDYLKKR--QSGSWMYLSRLAAMKEFAFMKALREEGFPV
AN0124.1  	TQRILKIHRLGRISFRTVKTNRDYLRNR--STGSWMYMSRLAAIKEFAFMKALRENGFSV
          	 *::************:**:*****::*    *****:*****:**:***:**:*:**.*

MG07119.1 	PEPLSQSRHTIVMSLIDAYPLRQISSVPNPAALYAELMLLILRLAKHGLIHGDFNEFNIL
NCU07722.1	PEPISQSRHTIVMSLVESLPLRQVSSVPDPASLYAELIDLILRLAKHGLIHGDFNEFNIL
FG09784.1 	PEPFAQSRHTIVMSLIDAFPLRQIAEVPDPASLYGDLIALILRLAKHGLIHGDFNEFNIL
AN0124.1  	PEPIAQNRHTIVMSLIDAFPLRQISKVPKPALLYAELMDTIMRLARYGLIHGDFNEFNIL
          	***::*.********::: ****::.**.** **.:*:  *:***::*************

MG07119.1 	LKEEKDKKNHASSTESAENGSSEVGAALPTPILIDFPQMVSMDHPNAEMYFDRDVACIKR
NCU07722.1	IKEERTTKYTIEDGEEKETE-----TVTLVPVIIDFPQMVSMDHVNAEMYFDRDVECIKR
FG09784.1 	IKENITKS---EEGEE---------TLTLEPVVIDFPQMVSMEHQNAEMYFDRDVNCVKR
AN0124.1  	IKEEVNPEAKGKGREGEDDDE----NLKLTPVIIDFPQMVSVDHPNAEMYFDRDVNCIKR
          	:**:   .   .  *  .  .         *::********::* ********** *:**

MG07119.1 	FFERRFHFSSDEPGPFYKDALKTVGADGARRLDATVEASGFTKKMSKDLEKAIQEEAASR
NCU07722.1	FFKKRFHFVPTQPGPFFKDAKKTVGKDGAKRLDATVEASGFTKKMLKELEKAIQEQQAKI
FG09784.1 	FFERRFHFVPTEPGPFFKHAKKAMTKEGLKRLDATVEASGFTKKMLKDLEAAIKEKGDET
AN0124.1  	YFQRKFHFVSDVPGPFFADAKKQLKKNPGKRLDVEVEASGFSRKMARELEKYMKEVGVDG
          	:*:::*** .  ****: .* * :  :  :***. ******::** ::**  ::*   . 

MG07119.1 	RE----QTNDIDGEFEGSDAEQDSDAEAESEQADLNGSGQTEHDASDSKLPLTG----IE
NCU07722.1	DAGEAVEEEELD-EDEDEDEEDDSGSDTDDEEDEEDQQEEEEHAVFEIPANVGRPLSDAE
FG09784.1 	TQ----QSDDED-DDDDEDEEDDEEGGSEDPKDGQTSGGLLGDDVLNSKDDP------VE
AN0124.1  	DN------GEAD--KEGAESDNEDGSGSEDDTAGQSETDTKEENSNRSLEES-------S
          	         : *   :. : :::. . ::.            .                .

MG07119.1 	AAVQGTKISDTS-----
NCU07722.1	KGLEKLSLGGKGGKRRA
FG09784.1 	ESMSKLAV---------
AN0124.1  	QKLEQLHVS--------
          	  :.   :.
```
